# Autism and Sensory Processing Disorders: Shared White Matter Disruption in Sensory Pathways but Divergent Connectivity in Social-Emotional Pathways

**DOI:** 10.1371/journal.pone.0103038

**Published:** 2014-07-30

**Authors:** Yi-Shin Chang, Julia P. Owen, Shivani S. Desai, Susanna S. Hill, Anne B. Arnett, Julia Harris, Elysa J. Marco, Pratik Mukherjee

**Affiliations:** 1 Department of Radiology and Biomedical Imaging, University of California San Francisco, San Francisco, California, United States of America; 2 Department of Neurology, University of California San Francisco, San Francisco, California, United States of America; University of Minnesota, United States of America

## Abstract

Over 90% of children with Autism Spectrum Disorders (ASD) demonstrate atypical sensory behaviors. In fact, hyper- or hyporeactivity to sensory input or unusual interest in sensory aspects of the environment is now included in the DSM-5 diagnostic criteria. However, there are children with sensory processing differences who do not meet an ASD diagnosis but do show atypical sensory behaviors to the same or greater degree as ASD children. We previously demonstrated that children with Sensory Processing Disorders (SPD) have impaired white matter microstructure, and that this white matter microstructural pathology correlates with atypical sensory behavior. In this study, we use diffusion tensor imaging (DTI) fiber tractography to evaluate the structural connectivity of specific white matter tracts in boys with ASD (n = 15) and boys with SPD (n = 16), relative to typically developing children (n = 23). We define white matter tracts using probabilistic streamline tractography and assess the strength of tract connectivity using mean fractional anisotropy. Both the SPD and ASD cohorts demonstrate decreased connectivity relative to controls in parieto-occipital tracts involved in sensory perception and multisensory integration. However, the ASD group alone shows impaired connectivity, relative to controls, in temporal tracts thought to subserve social-emotional processing. In addition to these group difference analyses, we take a dimensional approach to assessing the relationship between white matter connectivity and participant function. These correlational analyses reveal significant associations of white matter connectivity with auditory processing, working memory, social skills, and inattention across our three study groups. These findings help elucidate the roles of specific neural circuits in neurodevelopmental disorders, and begin to explore the dimensional relationship between critical cognitive functions and structural connectivity across affected and unaffected children.

## Introduction

The human brain is a sensory processor. Its core function is to perceive, integrate, interpret, and then facilitate the appropriate coordinated response to the visual, tactile, auditory, olfactory, and proprioceptive information present in the world around us. Thus it comes as no surprise that inaccurate or imprecise sensory processing and multisensory integration (MSI) can lead to impaired intellectual and social development [1]–[4]. There is a growing recognition of the crucial importance of sensory processing as it contributes to attention, learning, emotional regulation, and even social function in children affected by a wide spectrum of neurodevelopmental disorders, including autism. There is also a growing interest in studying sensory processing and cognition as dimensional traits across typically developing children and those with psychiatric labels such as autism.

Autism spectrum disorders (ASD) have traditionally been characterized by impaired communication, social interaction, and behavioral flexibility [5]. However, individuals with ASD have also been shown to have ubiquitous challenges in sensory processing [6] with over 90% of children with autism reported to have atypical sensory related behaviors. In fact, hyper- or hyporeactivity to sensory input or unusual interest in sensory aspects of the environment is now included in the current DSM 5 diagnostic criteria for ASD [6]. There are, however, children with sensory processing disorders (SPD) who do not show primary language or social deficits but do exhibit atypical sensory reactivity and/or sensory interests to the same or greater extent as children who meet an ASD diagnosis [1]. Children with SPD remain critically underserved with regard to their developmental challenges in our society due to the lack of a diagnostic label recognized in the current DSM 5 manual. Many are instead attributed labels that better describe the sequelae of SPD, such as oppositional defiant disorder, than the root of the problem. It is therefore highly relevant to better characterize the biological bases of this increasingly recognized neurodevelopmental condition. In addition, the comparison of children with SPD and ASD may help to illuminate the unique neural mechanisms at the core of the ASD diagnosis: those facilitating social awareness, interest, and drive. With over 1% of children in the USA carrying an ASD label and reports of 5–16% of children in the USA having sensory processing difficulties, it is important to define the neural underpinnings of these conditions and to delineate the areas of overlap and the areas of divergence [1], [2], [7]. The advent of diffusion tensor imaging (DTI) and fiber tractography has enabled quantitative, noninvasive evaluation of white matter microstructure and connectivity. There is considerable, albeit contradictory, literature reporting altered structural connectivity in individuals with ASD using DTI [8]. There are several studies suggesting reduced connectivity via the corpus callosum [9]–[11] as well as others indicating normal or even elevated fractional anisotropy (FA), a measure of white matter tract microstructural integrity from DTI [12]. Beyond the corpus callosum, there are also reports of other white matter tracts that may show variance from typically developing controls, including the inferior fronto-occipital fasciculus (IFOF) and the uncinate fasciculus (UF). A recent meta-analysis of 25 DTI studies in individuals with autism reports decreased FA in the corpus callosum, the left UF, and the left superior longitudinal fasciculus (SLF), supporting the theory of specific underconnectivity in autism focused on tracts supporting auditory information and language processing [13]. Finally, in addition to auditory and language related tracts, there is considerable interest in tracts that mediate emotional face recognition, a pervasive deficit in children with autism. DTI studies have specifically investigated the fusiform-hippocampal and fusiform-amygdala tracts in individuals with autism and have reported variation thought to relate to atypical function [14], [15].

In comparison to DTI studies of ASD, investigation of structural connectivity in children with isolated SPD is in its infancy. We recently reported that, although children with SPD do not exhibit morphological abnormalities from structural MR imaging, they have strikingly decreased white matter microstructural integrity, especially in posterior cerebral regions [16]. These regions are implicated in unimodal sensory processing as well as MSI, and are regulated by top-down attention modulation via thalamic projections. We further showed that white matter connectivity correlates with behavioral measures of unimodal sensory behavior, multisensory integration, and inattention. White matter microstructural integrity is crucial to the speed and bandwidth of information transmission throughout the brain. Degraded connectivity of primary sensory cerebral tracts or of pathways connecting multimodal sensory association areas may thereby result in the loss of the precise timing of action potential propagation needed for accurate sensory registration and integration. These effects may be reflected in assessable metrics such as processing speed and working memory, the latter of which has been proposed to be mediated by stereotypical time-locked spatiotemporal spike timing patterns [17].

In this study, we examine white matter tracts that we hypothesize will be atypical in children with SPD or ASD subjects relative to typically developing children (TDC). Based upon our previous work on white matter microstructure in SPD [16], and upon previous studies of white matter microstructure in ASD, we posit that both ASD and SPD subjects will have reduced structural connectivity compared to controls in parieto-occipital white matter tracts involved with sensory processing and integration, whereas only ASD subjects will have diminished structural connectivity relative to controls in temporal tracts associated with social-emotional processing. Furthermore, we posit that tract connectivity will correlate with measures reflecting sensory processing, inattention behavior, social behavior, verbal comprehension, processing speed, and working memory across groups.

## Methods

The Institutional Review Board (IRB) at the University of California in San Francisco approved this study (UCSF IRB Protocol #: 10-01940). Subjects were recruited from the UCSF Autism and Neurodevelopment Program clinical sites and research database, and from local online parent board listings. Informed consent was obtained from the parents or legal guardians, with the assent of all participants.

### 2.1. Demographic, sensory, cognitive and behavioral data

#### 2.1.1. General demographics

Sixteen right-handed males with SPD, fifteen males with ASD (12 right-handed, 1 left-handed, 2 ambidextrous), and 23 right-handed male TDC, all between 8 and 12 years of age, were prospectively enrolled under our IRB protocol.

Voxel-based analysis of the DTI data from the 16 SPD subjects and the 23 TDC using tract-based spatial statistics (TBSS) to investigate white matter microstructure was previously reported in [16]. Group differences in the TBSS analysis were determined in a common atlas space after inter-subject image registration. In the present study, we examine white matter connectivity using diffusion fiber tractography in each subject’s native space, with the addition of an ASD cohort.

#### 2.1.2. General cognition

All subjects were assessed with the Wechsler Intelligence Scale for Children-Fourth Edition [18] and were required to have a Perceptual Reasoning Index (PRI) score ≥70. We used PRI as our measure of cognition for inclusion, as communication deficits are part of the core diagnosis of ASD. Verbal Comprehension Index (VCI), Processing Speed Index (PSI), and Working Memory Index (WMI) were also obtained from this assessment. These measures are displayed in Table 1 for each cohort.

**Table 1:** Cognitive Characterization of TDC, ASD, and SPD Cohorts.

	TDC Mean ± Std	ASD Mean ± Std	Pval	SPD Mean ± Std	Pval
PRI	113.5±13.5	101.6±14.1	**0.015**	115.8±11.5	0.576
VCI	119.2±12.7	101.6±20.5	**0.007**	117.4±12.8	0.660
WMI	108.4±10.9	99.6±17.7	0.111	104.4±12.8	0.320
PSI	101.3±13.6	87.4±11.1	**0.002**	97.1±12.9	0.334

PRIs, VCIs, WMIs and PSIs for each cohort, with p values from two-tailed t-tests for differences between TDCs and each patient cohort (statistically significant p values of less than 0,05 are indicated in boldface).

#### 2.1.3. Sensory processing evaluation

All subjects were evaluated with the Sensory Profile [19], which is currently the most widely used parent report measure of atypical sensory related behavior. The Sensory Profile (SP) is a caregiver report questionnaire (125 items) which measures behavioral sensory differences, yielding scores within individual sensory domains and factors as well as a total score. A probable difference (PD) in sensory behavior is defined as a total score between 142 and 154, while a definite difference (DD) is a score of ≤141. Lower scores reflect more atypical behavior. We use the auditory, visual, tactile, multisensory integration, and inattention/distractibility scores to explore behavioral correlations based on findings from our prior report [16].

Inclusion in the SPD group required a community based Occupational Therapy diagnosis of Sensory Processing Disorder plus a score in the definite difference (DD) range, defined as greater than two standard deviations from the mean, of either the total or the auditory processing score of the Sensory Profile. Five of the SPD subjects scored in the DD range for total score alone, four scored in the DD range for the auditory processing score alone, and seven scored in the DD range for both the total and auditory score. Two ASD subjects scored in the DD range for the total score alone, one ASD subject scored in the DD range for the auditory score alone, and seven of the ASD subjects scored in the DD range for both the total and auditory score. The sensory profile was not obtained for one ASD individual. All of the controls scored in the normal range (Table 2).

**Table 2:** Sensory Profile Characterization of TDC, ASD, and SPD cohorts.

	TDC Mean ±Std	ASD Mean ±Std	SPD Mean ±Std
Auditory	33.6±3.5	*24.4±5.9	*22.7±4.9
Tactile	83.3±5.8	72.4±8.6	*62.9+8.8
Visual	41.2±3.0	35.6±6.3	32.3±7.1
Inattention	28.7±3.6	*20.3±4.4	*17.8±5.3
Total	172.3±11.0	*135.1±18.2	*128.5±15.8
Multisensory	31.3±3.1	23.7±4.5	22.2±3.7

Asterisks indicate mean scores that fall within the definite difference range. None of the mean scores fell in the probable difference range.

#### 2.1.4. Autism evaluation

All subjects were evaluated with the Social Communication Questionnaire (SCQ), a parent report ASD screening instrument [20]. All of the ASD cohort (carrying community diagnosis of ASD) as well as the SPD individuals with a score above threshold (≥15) were evaluated with the Autism Diagnostic Inventory-Revised (ADI-R) [21], a parent history interview, and the Autism Diagnostic Observation Schedule (ADOS) [22], a structured play session. We used current diagnostic scoring for the ADOS and lifetime scoring for the ADI-R. None of the TDC cohort had an SCQ score ≥15. All participants in the ASD cohort met criteria on both the ADI-R and ADOS; all but one scored ≥15 on the SCQ.

Three of the SPD cohort scored above 15 on the SCQ and were further evaluated with the ADI-R and ADOS. One SPD participant scored above the ASD cutoff on the current diagnosis scoring of the ADOS but did not meet criteria on the ADI-R. Another SPD individual met criteria on the ADI-R but not the ADOS. Neither was considered to meet clinical criteria when evaluated by a cognitive behavioral child neurologist with expertise in autism and neurodevelopment (EJM). The third SPD participant who scored above 15 on the SCQ met neither the ADI-R nor ADOS cut-off. A supplementary analysis was performed, excluding these three SPD subjects from the study cohort (Table S2).

#### 2.1.5. Attention deficits

On the inattention/distractibility factor of the Sensory Profile, eleven of the 16 SPD subjects scored in the definite difference range, four in the probable difference range, and one in the typical range. Of the 15 ASD subjects, seven scored in the definite difference range, five scored in the probable difference range, two scored in the typical range, and one was not administered the Sensory Profile. Of the 23 TDC, none scored in the definite difference range, three in the probable difference range, and twenty in the typical range. Atypical inattention/distractibility scores on the Sensory Profile do not necessarily indicate that individuals would meet clinical criteria for an attention deficit (hyperactivity) disorder (ADHD) diagnosis. Formal ADHD evaluations were not conducted as part of this study.

#### 2.1.6. Prematurity

Three of 16 SPD boys were born prematurely, one at 32 weeks gestation and two at 34 weeks gestation. One of the 23 typically developing children was born prematurely, at 33 weeks gestation. These four subjects were found to be in the middle of the distribution for global FA and mean FA extracted from clusters of significantly affected voxels using TBSS for their respective groups, and therefore they were not considered to be outliers [16]. None of the ASD subjects were born prematurely.

### 2.2. Image acquisition

MR imaging was performed on a 3T Tim Trio scanner (Siemens, Erlangen, Germany) using a 12-channel head coil. Structural MR imaging of the brain was performed with an axial 3D magnetization prepared rapid acquisition gradient-echo (MP-RAGE) T1-weighted sequence (TE = 2.98 ms, TR = 2300 ms, TI = 900 ms, flip angle of 9°) with a 256 mm field of view (FOV), and 160 1.0 mm contiguous partitions at a 256×256 matrix. Whole-brain DTI was performed with a multislice 2D single-shot twice-refocused spin-echo echo-planar sequence with 64 diffusion-encoding directions, diffusion-weighting strength of b = 2000 s/mm^2^, iPAT reduction factor of 2, TE/TR = 109/8000 ms, averages = 1, interleaved 2.2 mm axial slices with no gap, and in-plane resolution of 2.2×2.2 mm with a 100×100 matrix and FOV of 220 mm. An additional volume was acquired with no diffusion weighting (b = 0 s/mm^2^). The total DTI acquisition time was 8.67 min.

### 2.3. DTI analysis

#### 2.3.1. Pre-processing

The diffusion-weighted images were corrected for motion and eddy currents using FMRIB’s Linear Image Registration Tool (FLIRT; www.fmrib.ox.ac.uk/fsl/flirt) with 12-parameter linear image registration [23]. All diffusion-weighted volumes were registered to the reference b = 0 s/mm^2^ volume. To evaluate subject movement, we calculated a scalar parameter quantifying the transformation of each diffusion volume to the reference. A heteroscedastic two-sample Student’s t-test verified that there were no significant differences between SPD, ASD, and TDC groups in movement during the DTI scan (p>0.05). The non-brain tissue was removed using the Brain Extraction Tool (BET; http://www.fmrib.ox.ac.uk/analysis/research/bet). FA was calculated using the FMRIB Software Library (FSL) DTIFIT function.

#### 2.3.2. High angular resolution diffusion imaging (HARDI) and fiber tractography

The FSL bedpostx tool was used for HARDI reconstruction of the diffusion data, modeling multiple fiber orientations per voxel, and thereby accounting for crossing fibers [24]. Probabilistic streamline tractography was performed using FSL’s probtrackx2 to delineate white matter tracts of interest, using the strategies described in Tables 3–5 and illustrated in Figure S1. Seed, waypoint, termination, and exclusion masks for tractography were primarily derived from the gray-white matter boundaries (GWB) of the 82 Freesurfer cortical and subcortical regions, which were automatically segmented on the T1-weighted MR images using Freesurfer 5.1.0 [25] and registered using a linear affine transformation to diffusion space using FLIRT. The left and right cerebral peduncles were manually defined for each subject.

**Table 3:** Tractographical approach for temporal tracts.

White matter tract	Seed mask	Waypoint and termination mask	Exclusion mask
Fusiform - amygdala	Fusiform gyrus	Amygdala	All other gm regions
Fusiform - hippocampus	Fusiform gyrus	Hippocampus	All other gm regions
Uncinate fasciculus (UF)	Orbitofrontal cortex*	Entorhinal cortex + temporal pole	All other gm regions
Inferior longitudinal fasciculus (ILF)	Pericalcarine cortex	Inferior temporal cortex	Thalamus + all other cortical regions
Inferior frontooccipital fasciculus (IFOF)	Lingual gyrus	Orbitofrontal cortex*	Thalamus + all other cortical regions

The Freesurfer seed, waypoint, termination, and exclusion masks used in fiber tractography to delineate examined temporal tracts. *Orbitofrontal cortex was created by summing the medial orbitofrontal cortex and lateral orbitofrontal cortex.

**Table 4:** Tractographical approach for parieto-occipital tracts.

White matter tract	Seed mask	Waypoint and termination mask	Exclusion mask
Optic radiation	Pericalcarine cortex	Eroded thalamus	All other cortical regions
Dorsal visual stream	Pericalcarine cortex	Inferior parietal cortex	Thalamus
Splenium of the corpus callosum	Left lateral occipital cortex	Right lateral occipital cortex*	All other cortical regions
Posterior corona radiata (PCR) (occipital)	All occipital regions	Cerebral peduncle	All other cortical regions
Posterior corona radiata (PCR) (parietal)	All parietal regions	Cerebral peduncle	All other cortical regions

The Freesurfer seed, waypoint, termination, and exclusion masks used in fiber tractography to delineate examined parieto-occipital tracts. *For the tract through the splenium of the corpus callosum, a callosal waypoint mask was also used.

**Table 5:** Tractographical approach for frontal tracts.

White matter tract	Seed mask	Waypoint and termination mask	Exclusion mask
Anterior thalamic radiation(ATR) (medial orbitofrontal cortex)	Medial orbitofrontal cortex	Eroded thalamus	All other gm regions
Anterior thalamic radiation(ATR) (rostral middle frontal cortex)	Rostral middle frontal cortex	Eroded thalamus	All other gm regions
Genu of the corpus callosum(medial orbitofrontal cortex)	Left medial orbitofrontal cortex	Right medial orbitofrontal cortex	All other cortical regions
Genu of the corpus callosum(rostral middle frontal cortex)	Left rostral middle frontal cortex	Right rostral middle frontal cortex	All other cortical regions
Anterior corona radiata (ACR)	All frontal regions	Cerebral peduncle	All other cortical regions

The Freesurfer seed, waypoint, termination, and exclusion masks used in fiber tractography to delineate examined frontal tracts. *For the tracts through the genu of the corpus callosum, a callosal waypoint mask was also used.

#### 2.3.3 Tract delineation

Subsequent to performance of probabilistic streamline fiber tractography, tract masks for every tract described in Tables 3–5 were separately generated for each subject. Each mask was created by taking the intersection of the binarized, thresholded, tractography-derived streamline map and a binary mask of FA>0.2. The streamline threshold used for binarization was separately calculated for each streamline map, and equal to 1% multiplied by the maximum number of streamlines passing through any voxel in the map. This streamline threshold was a consistent strategy of removing spurious streamlines, while retaining most voxels contained within the desired white matter tract. The FA threshold further ensured that the voxels contained within the mask were confined to white matter. Additionally, each tract mask for each subject was visually inspected to confirm that the anatomy of each target tract was accurately and consistently defined.

White matter connectivity was calculated as the average FA value within the delineated tract of interest. This measurement has been shown to be highly reproducible in cross-sectional [26] and longitudinal studies [27].

**Figure 1:**
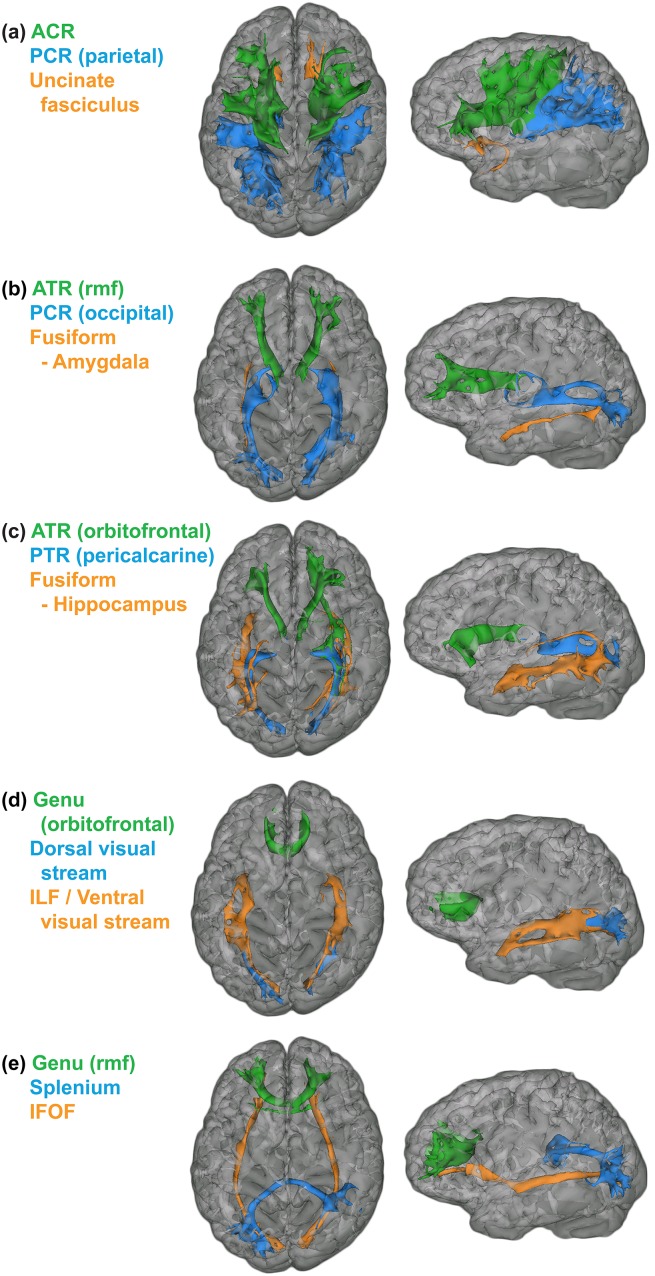
Examples of each delineated tract for a representative subject. Green masks represent frontal tracts, blue masks represent parietal-occipital tracts, and orange masks represent temporal tracts. The tracts are superimposed upon the T1 image, registered to diffusion space and with decreased opacity, of the representative subject.

Representative examples of each of the 15 delineated tracts are displayed in Figure 1. All masks used for tractography were the GWBs of Freesurfer regions except for manually-defined cerebral peduncles and corpus callosum masks. Eroded thalamus masks refer to an eroded version of the Freesurfer thalamus which was transformed using the *fslmaths* erode filtering operation with a box kernel of width 9, a step taken to prevent the thalamic mask from overlapping the corpus callosum and resulting in spurious interhemispheric streamlines. Except for callosal connections, each tract was delineated separately in both the left and right hemispheres. Following mask extraction (after thresholding by streamlines and FA), corresponding left and right hemisphere tract masks were combined for subsequent analysis. The unilateral tracts were individually assessed to confirm bilateral consistency and to evaluate hypothesized tract laterality.

### 2.4. Statistical analysis of group differences

For each tract, decreases in FA were separately assessed for the SPD and ASD cohorts relative to controls using one-tailed permutation tests (n = 10,000) (adapted from [28]). Permutation testing was utilized, as it is a nonparametric method and thereby does not assume normally distributed data. The true two-sample t statistic was calculated for control FA vs. patient FA, and a two-sample t statistic distribution was generated by permuting the control and patient labels 10,000 times, calculating a t-statistic value each time. The one-tailed p value was then calculated as the number of permuted t statistic values lying below the true t statistic, divided by the number of permutations (10,000). Group differences were assessed separately for each patient cohort relative to controls at a false discovery rate (FDR) - corrected p value threshold (from p<0.05), with FDR correction applied separately to tracts within each region (separately for the temporal, parietal-occipital, and frontal tracts). Because the perceptual reasoning index (PRI) scores were significantly lower for the ASD cohort compared to the TDC and SPD subjects, a post-hoc group difference analysis was conducted while controlling for PRI. For each tract, a general linear model (GLM) was fit to the data using PRI as a regressor, and permutation tests were performed in the same way as described above, using t statistics for the group coefficient estimates from the GLM. Differences were again assessed using FDR correction within the temporal, parietal-occipital, and frontal regions.

### 2.5. Cognitive associations

Pearson’s correlations of FA in the 15 examined tracts with the VCI, PRI, WMI, PSI, the social component of the SCQ, and the five subtests of the SP (auditory, visual, tactile, inattention, multisensory integration) were investigated dimensionally across all individuals. Statistical significance was assessed at p<0.05 with FDR correction across all 15 tracts. For tracts and cognitive/behavioral metrics demonstrating significant associations across groups, post-hoc correlational analyses were conducted for the unilateral tract FA (left and right hemisphere independently) across groups, as well as unilateral and bilateral tract FA (left and right combined) for each cohort (TDC, SPD, and ASD) independently.

## Results

### 3.1. Group differences in white matter connectivity

**Figure 2:**
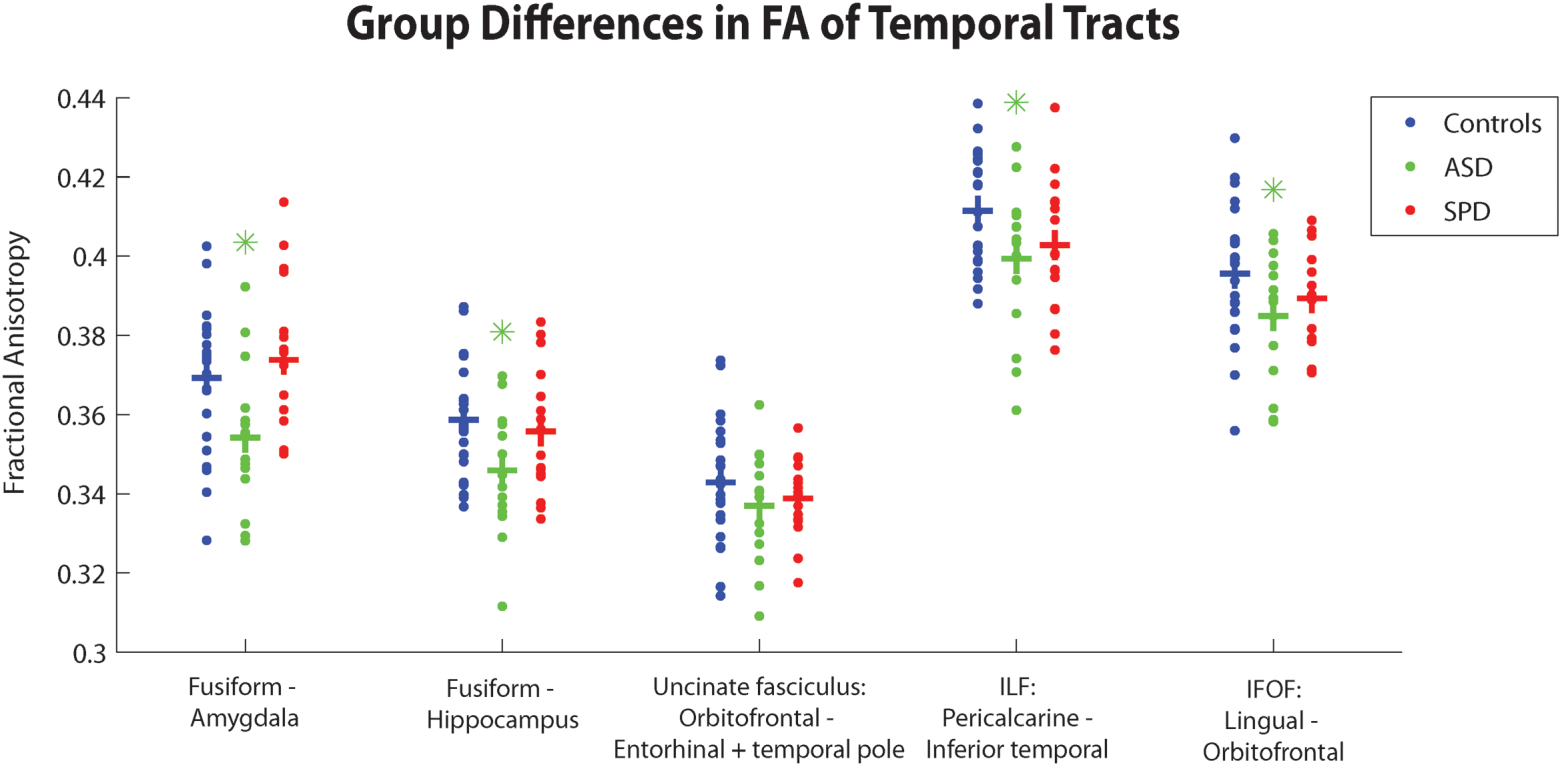
Group differences between TDC, SPD, and ASD subjects in average FA within different temporal tracts. Crossbars correspond to group averages. Green asterisks depict significant group differences between ASD and TDC subjects, and red asterisks depict significant group differences between SPD and TDC subjects, FDR corrected at p<0.05.

**Figure 3:**
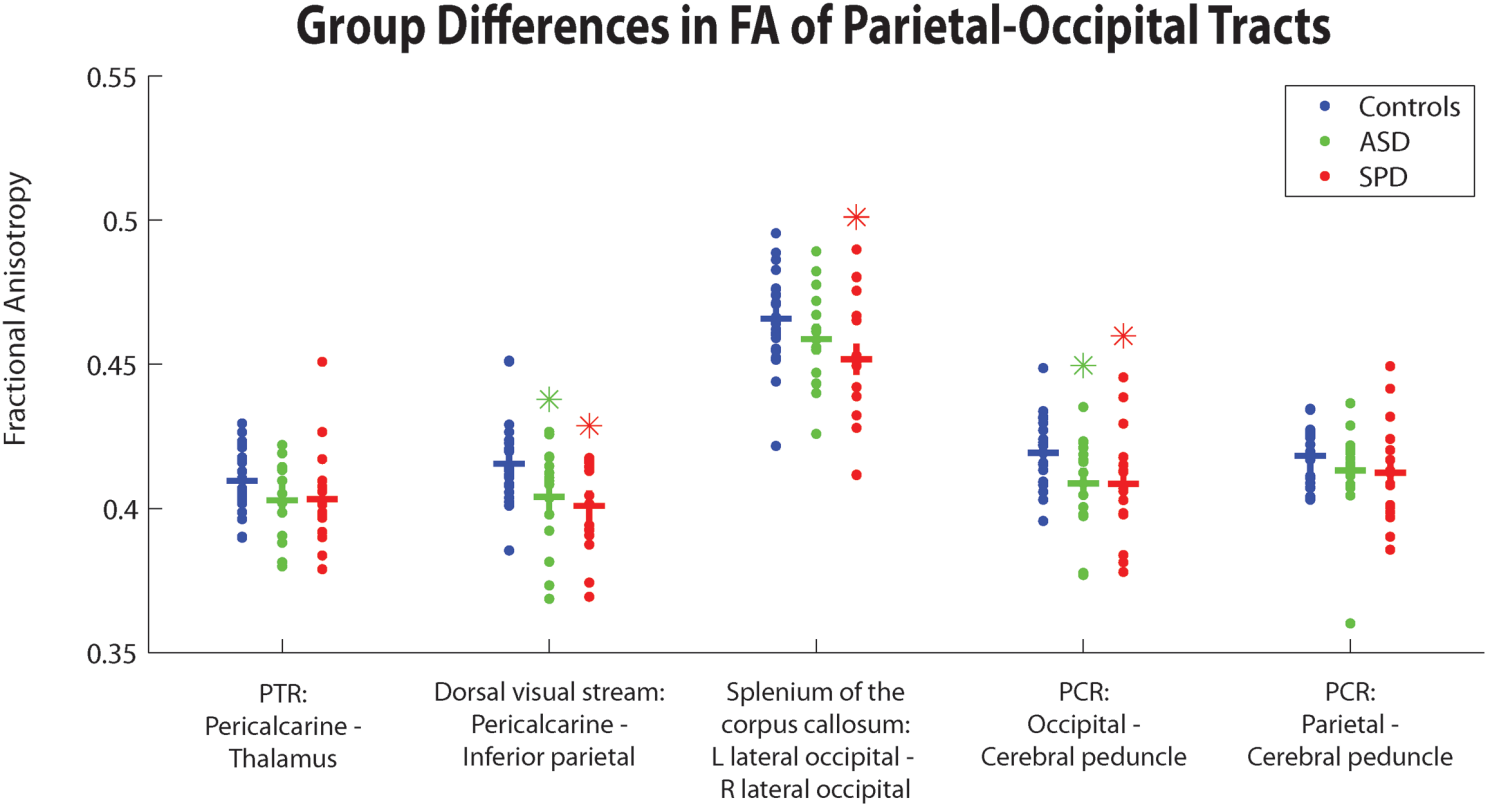
Group differences between TDC, SPD, and ASD subjects in average FA within different parietal-occipital tracts. Crossbars correspond to group averages. Green asterisks depict significant group differences between ASD and TDC subjects, and red asterisks depict significant group differences between SPD and TDC subjects, FDR corrected at p<0.05.

**Figure 4:**
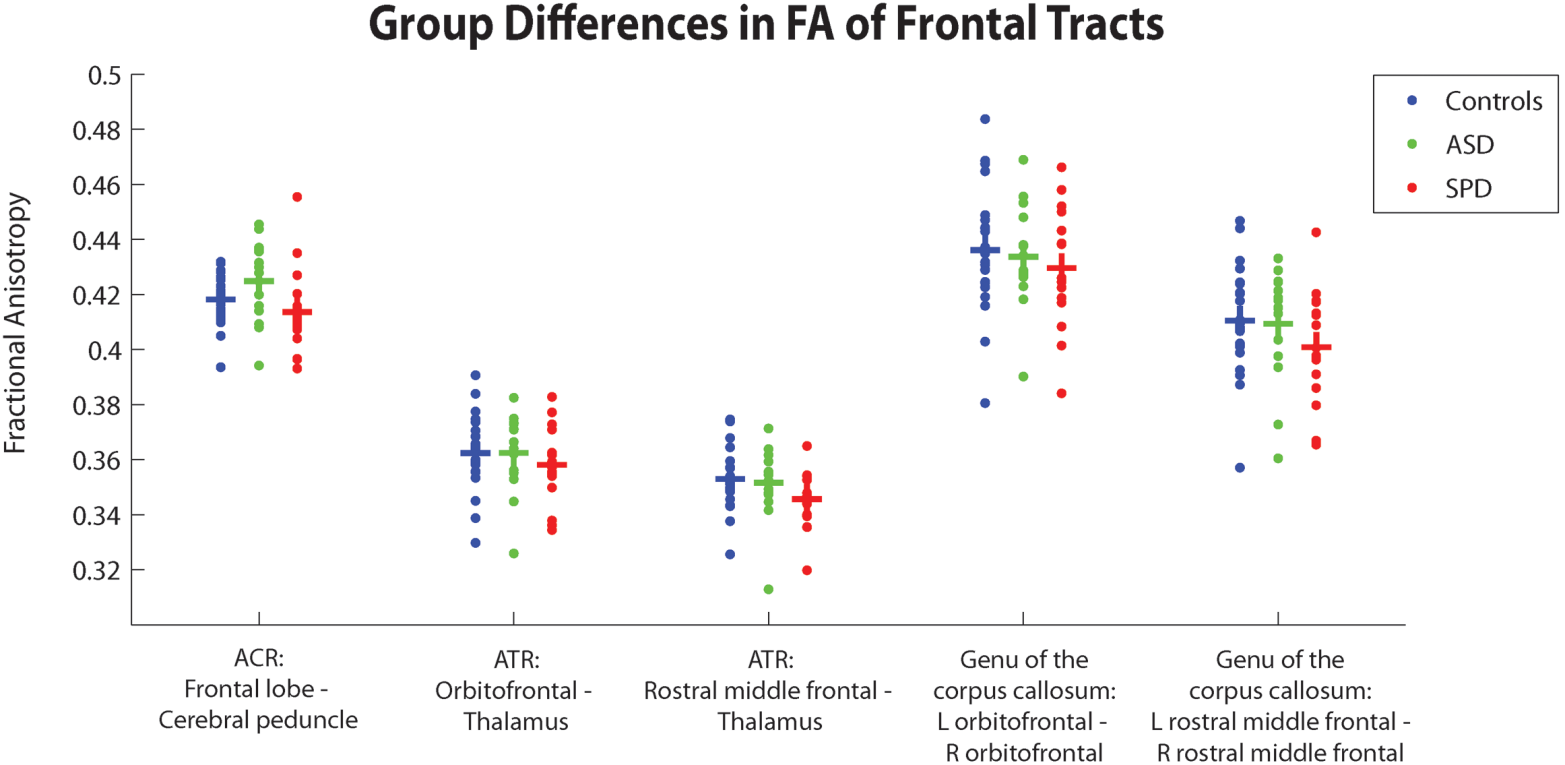
Group differences between TDC, SPD, and ASD subjects in average FA within different frontal tracts. Crossbars correspond to group averages. Green asterisks depict significant group differences between ASD and TDC subjects, and red asterisks depict significant group differences between SPD and TDC subjects, FDR corrected at p<0.05.

Figures 2–4 depict group differences of structural connectivity denoted by fractional anisotropy (FA) in tracts predominantly involving the temporal, parietal-occipital, or frontal regions. Table 6 quantitatively details these group differences. We have additionally added the results of mean diffusivity, axial diffusivity, and radial diffusivity for the three groups).

**Table 6:** Connectivity (FA) in all tracts.

Tract	TDC mean FA±SD	ASD mean FA±SD	P-val	SPD mean FA±SD	P-val
**Fusiform - amygdala**	0.3693±0.0182	0.3546±0.0177	**0.0112**	0.3738±0.0203	0.2298
**Fusiform - hippocampus**	0.3587±0.0146	0.3469±0.0156	**0.0078**	0.3558±0.016	0.282
**Uncinate fasciculus**	0.3429±0.0156	0.3375±0.0137	0.118	0.3388±0.0099	0.1886
**ILF**	0.4114±0.0147	0.4008±0.0192	**0.019**	0.4028±0.0164	0.0468
**IFOF**	0.3956±0.0171	0.3844±0.0156	**0.031**	0.3893±0.0128	0.11
**PTR (optic radiations)**	0.4095±0.0113	0.4029±0.0127	0.0516	0.4032±0.0175	0.0814
**Dorsal visual stream**	0.4155±0.0147	0.4052±0.0179	**0.0134**	0.4009±0.0156	**0.0028**
**Splenium of the CC** **(lat occipital)**	0.4658±0.0161	0.4589±0.0167	0.1012	0.4517±0.0238	**0.0164**
**PCR (occipital)**	0.4194±0.0123	0.4093±0.0161	**0.0144**	0.4085±0.019	**0.0198**
**PCR (parietal)**	0.4182±0.0093	0.4142±0.0168	0.1338	0.4124±0.0178	0.1018
**ACR**	0.4182±0.0091	0.4259±0.0146	0.0508	0.4136±0.0156	0.1236
**ATR (orbitofrontal)**	0.3623±0.0137	0.3627±0.0137	0.4984	0.3581±0.014	0.1694
**ATR (rostral middle frontal)**	0.3530±0.0111	0.3532±0.0143	0.3616	0.3457±0.0105	0.0238
**Genu of the CC (orbitofrontal)**	0.4361±0.0224	0.4338±0.0179	0.3666	0.4296±0.0218	0.1916
**Genu of the CC** **(rostral middle frontal)**	0.4105±0.0196	0.4078±0.0208	0.4306	0.4008±0.0204	0.0674

The mean and standard deviation of FA within each tract for each group, with associated p values for group differences of the TDC cohort with either the SPD cohort or the ASD cohort. Bolded p values represent significant group differences at p<0.05, FDR corrected.

#### 3.1.1. Group differences of connectivity in temporal tracts

Significantly impaired connectivity (lower FA) was detected for the ASD cohort alone relative to the TDC cohort in the fusiform-amygdala and fusiform-hippocampus tracts, the inferior fronto-occipital fasciculi (IFOF), and the inferior longitudinal fasciculi (ILF) (p<0.05, FDR corrected). The SPD cohort showed no significant differences in these tracts relative to the TDC cohort. There was no significant difference in connectivity of the uncinate fasciculi of either the ASD or SPD cohorts relative to the controls.

#### 3.1.2. Group differences of connectivity in parietal-occipital tracts

The SPD group alone showed significantly decreased connectivity in the splenium of the corpus callosum relative to the TDC cohort. Both the SPD and the ASD group showed reduced connectivity relative to controls in the dorsal visual stream and the posterior corona radiata (occipital portion) (all results with p<0.05, FDR corrected).

Neither the SPD nor ASD groups demonstrated significant differences in the optic radiations (pericalcarine – thalamus PTR) or parietal PCR relative to TDC; however, there were strong trends toward lower connectivity of the optic radiations in both the ASD and SPD groups relative to TDC.

#### 3.1.3. Group differences of connectivity in frontal tracts

Connectivity in the frontal tracts was not significantly decreased for either the SPD or ASD cohorts, although the SPD group showed trends towards decreased connectivity for all measured frontal tracts.

### 3.2. Unilateral versus bilateral white matter tracts

Homologous white matter tracts of the left and right cerebral hemispheres were combined for purposes of consolidation and improved statistical power. However, group differences were also computed unilaterally for each tract. In all cases, the results from bilateral tracts shown here agree with trends or statistically significant group differences from the component unilateral tracts, with no appreciable hemispheric asymmetries found.

### 3.3. Accounting for perceptual reasoning index variation

PRI was significantly lower in the ASD cohort compared to the TDC subjects, thus group differences in connectivity were computed while controlling for PRI scores. After controlling for PRI and including FDR correction, connectivity for the ASD cohort was no longer significantly lower in the IFOF or ILF, but still demonstrated decreased FA in the fusiform -amygdala and fusiform –hippocampus tracts. The results for the SPD subjects were unchanged when controlling for PRI, as expected since these subjects did not demonstrate differences in PRI relative to TDC.

### 3.4. Cognitive associations

**Figure 5:**
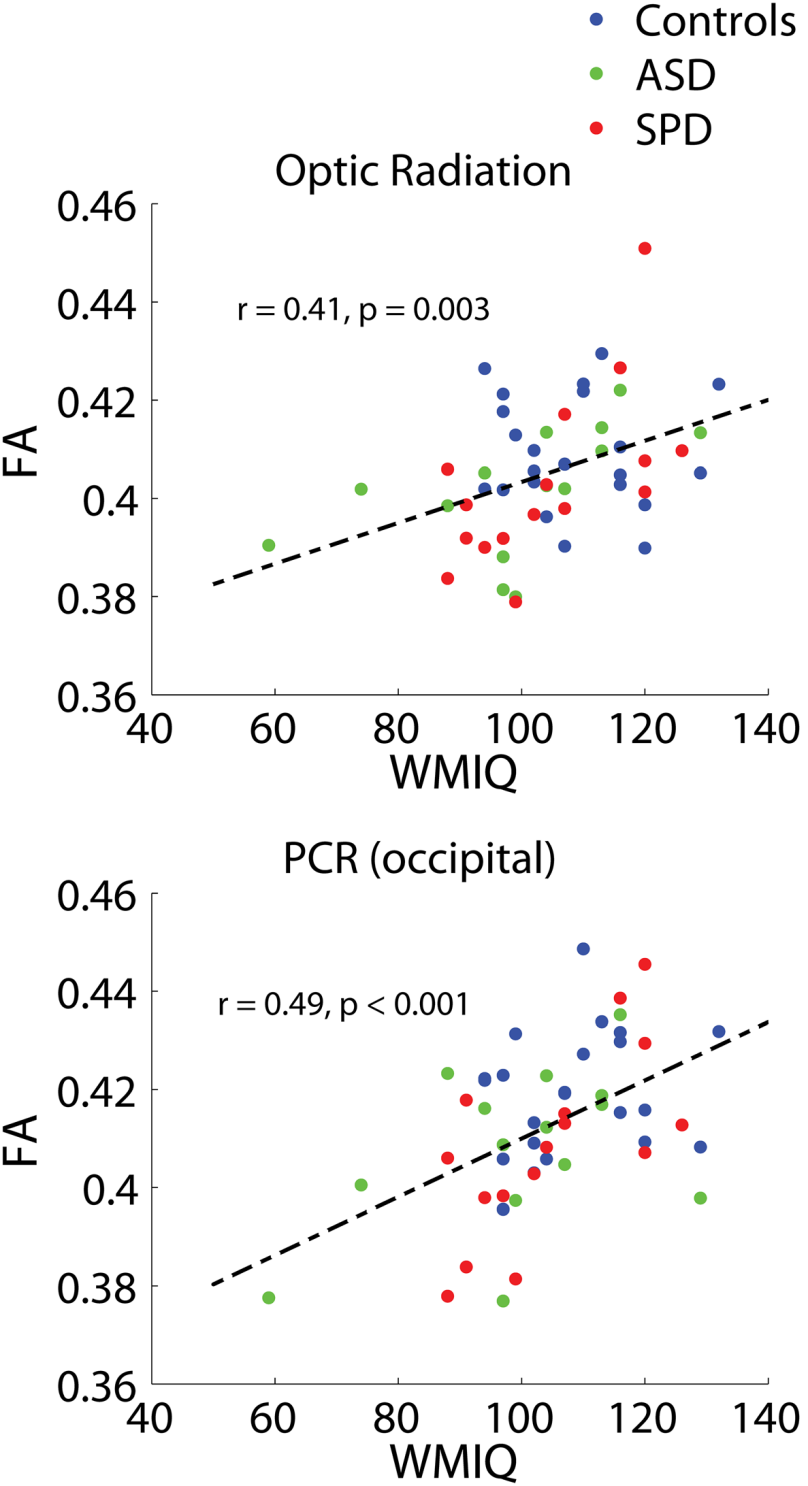
Combined-group associations between tract connectivity and WMI. The two bilateral tracts demonstrating significant associations between FA and WMI after FDR correction are displayed. Optic radiation: r = 0.41, p = 0.003. PCR (occipital): r = 0.49, p<0.001. Results of unilateral and individual group correlations are displayed in Table 7.

Significant combined-group correlations were found between WMI and the bilateral optic radiations (r = 0.41, p = 0.003) as well as the bilateral PCR (occipital) (r = 0.49, p<0.001) (Figure 5). These tracts both demonstrate left lateralized associations for the combined groups. The SPD cohort alone demonstrates significant individual-group associations between WMI and FA in both of these bilateral tracts, while ASD demonstrates significant or trend-level associations (Table 7).

**Table 7:** Associations between tract connectivity and WMI, for significantly associated tracts.

	r - bilateral	*p - bilateral*	r - left	*p - left*	r - right	*p - right*
PTR (optic radiation)						
All	0.41	***0.003***	0.36	***0.009***	0.32	***0.020***
TDC	−0.08	*0.715*	*0.07*	*0.753*	−*0.14*	*0.522*
ASD	0.54	***0.048***	*0.53*	*0.054*	*0.25*	*0.380*
SPD	0.62	***0.010***	0.35	*0.188*	0.73	***0.001***
PCR (occipital)						
All	0.49	***<0.001***	0.50	***<0.001***	0.32	***0.022***
TDC	0.22	*0.320*	0.39	*0.077*	−0.07	*0.746*
ASD	0.46	*0.101*	0.40	*0.161*	0.30	*0.299*
SPD	0.67	***0.004***	0.68	***0.004***	0.56	***0.024***

The bilateral tracts demonstrating significant combined-group associations with WMI are displayed. Correlation coefficients and p values are displayed for these tracts, along with combined group unilateral associations, individual group bilateral associations, and individual group unilateral associations.

**Figure 6:**
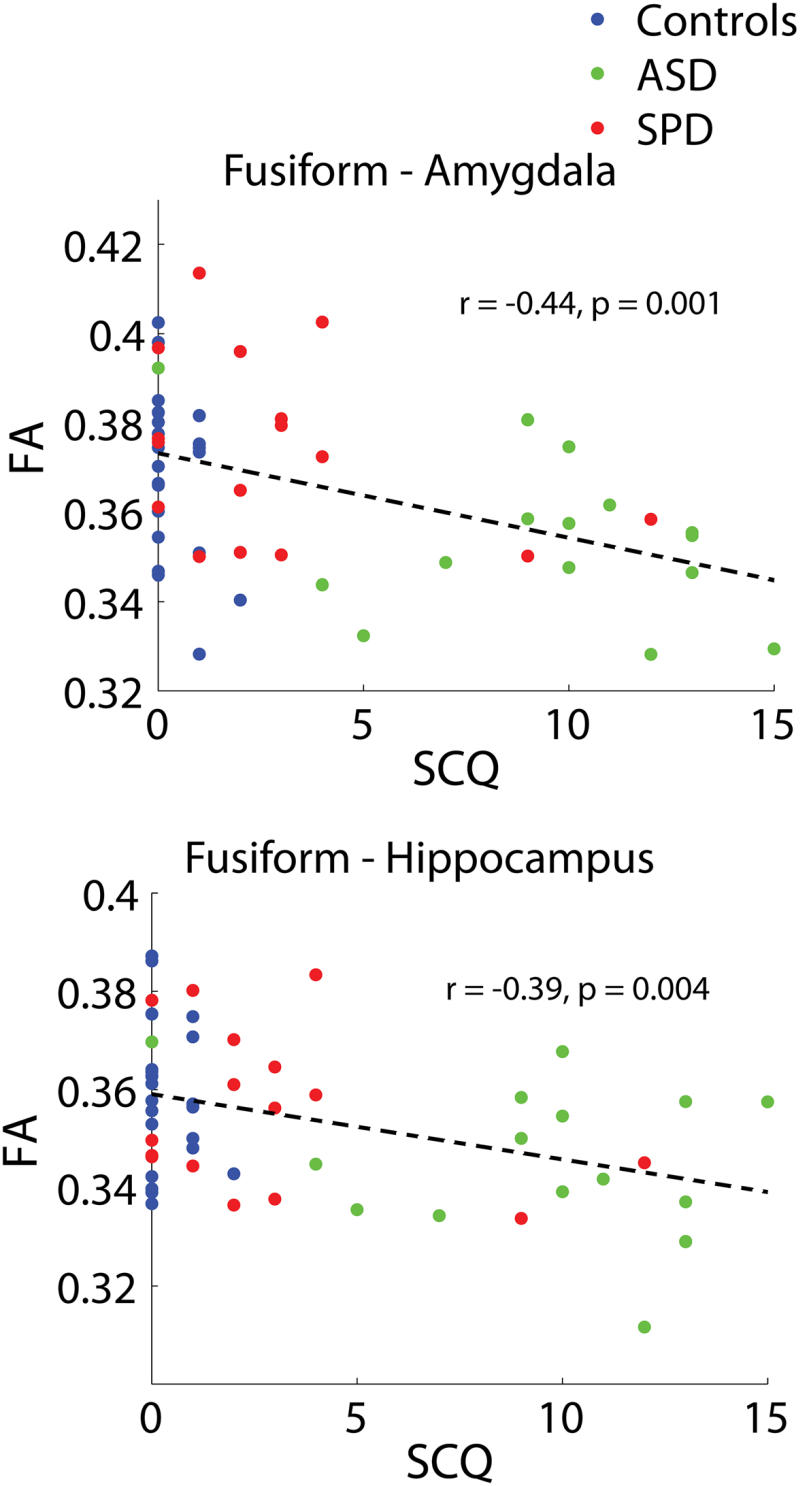
Combined-group associations between tract connectivity and SCQ-social. The two bilateral tracts demonstrating significant associations between FA and the social component of the SCQ after FDR correction are displayed. Fusiform-amygdala: r = −0.44, p<0.001. Fusiform-hippocampus: r = −0.39, p = 0.004. Results of unilateral and individual group correlations are displayed in Table 8.

Significant combined-group correlations were found between the social component of the SCQ and the bilateral fusiform - amygdala (r = −0.44, p = 0.001) as well as the bilateral fusiform-hippocampus (r = −0.39, p = 0.004) (Figure 6). These tracts both demonstrate right lateralized associations for the combined groups (Table 8).

**Table 8:** Associations between tract connectivity and the SCQ-social, for significantly associated tracts.

	r - bilateral	*p - bilateral*	r - left	*p - left*	r - right	*p - right*
Fusiform-amygdala						
All	−0.44	***0.001***	−0.27	***0.048***	−0.37	***0.006***
TDC	−0.39	*0.066*	−0.18	*0.423*	−0.29	*0.178*
ASD	−0.40	*0.137*	−0.34	*0.220*	−0.18	*0.515*
SPD	−0.32	*0.233*	0.03	*0.907*	−0.50	***0.049***
Fusiform-hippocampus						
All	−0.39	***0.004***	−0.25	*0.065*	−0.40	***0.003***
TDC	−0.14	*0.510*	−0.11	*0.613*	−0.15	*0.508*
ASD	−0.25	*0.370*	−0.22	*0.433*	−0.12	*0.680*
SPD	−0.27	*0.307*	−0.01	*0.979*	−0.47	*0.069*

The bilateral tracts demonstrating significant combined-group associations with the social component of the SCQ are displayed. Correlation coefficients and p values are displayed for these tracts, along with combined group unilateral associations, individual group bilateral associations, and individual group unilateral associations.

**Figure 7:**
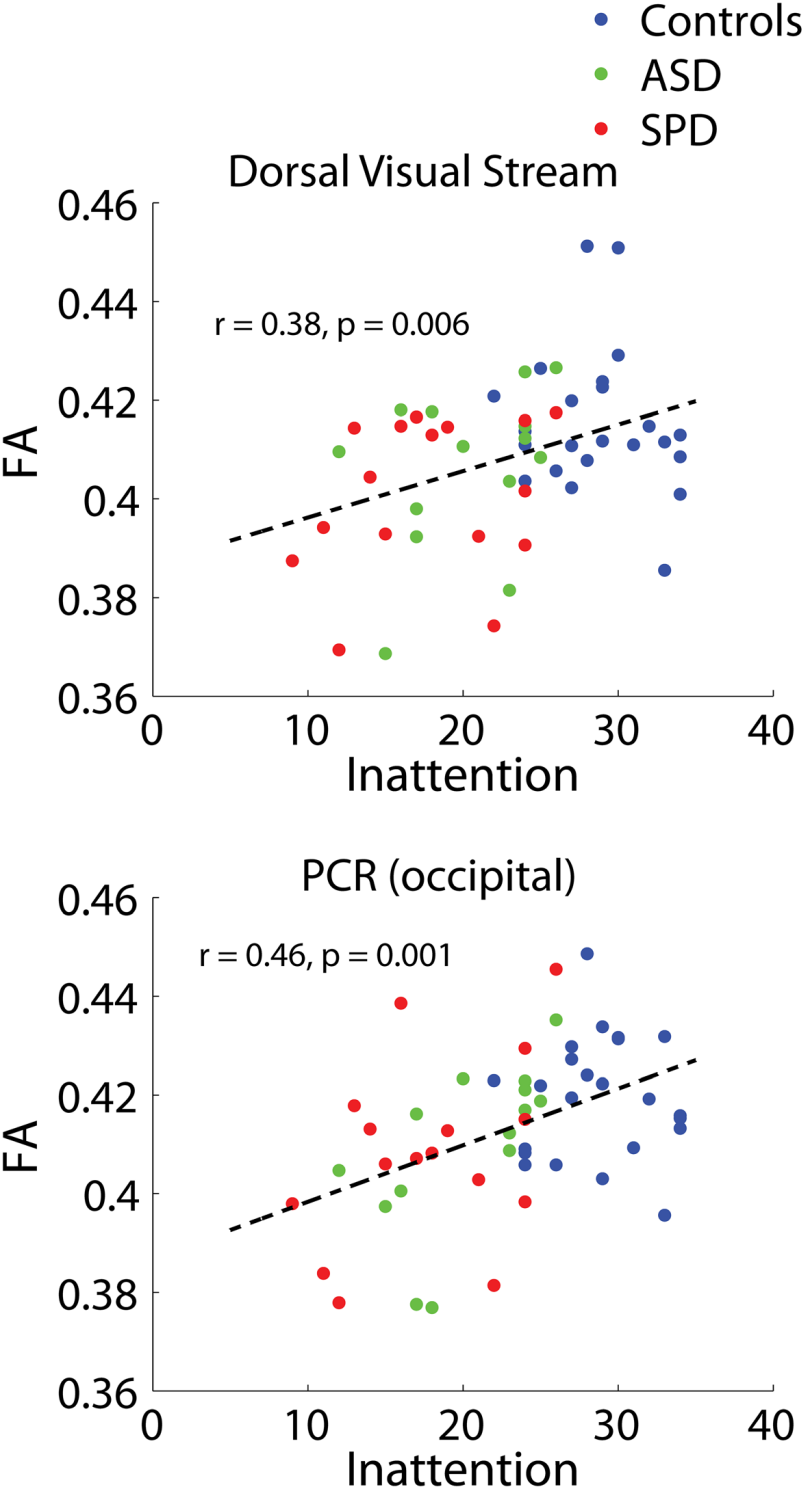
Combined-group associations between tract connectivity and the inattention measure of the Sensory Profile. The two bilateral tracts demonstrating significant associations between FA and the inattention measure of the Sensory Profile after FDR correction are displayed. Dorsal visual stream: r = 0.38, p = 0.006. PCR (occipital): r = 0.46, p<0.001. Results of unilateral and individual group correlations are displayed in Table 9.

Significant combined-group correlations were found between the inattention measures of the Sensory Profile and the dorsal visual stream (r = 0.38, p = 0.006) as well as the bilateral PCR (occipital) (r = 0.46, p = 0.001) (Figure 7). There is no strong evidence of lateralization for the combined groups (Table 9). The association between inattention and FA in the bilateral PCR (occipital) is consistent with our previously published findings in the combined SPD and TDC cohorts [16].

**Table 9:** Associations between tract connectivity and the inattention factor of the Sensory Profile, for significantly associated tracts.

	r - bilateral	*p - bilateral*	r - left	*p - left*	r - right	*p - right*
Dorsal Visual Stream						
All	0.38	***0.006***	0.34	***0.012***	0.27	*0.052*
TDC	−0.16	*0.479*	−0.09	*0.688*	−0.19	*0.408*
ASD	0.37	*0.197*	0.04	*0.885*	0.37	*0.195*
SPD	0.25	*0.358*	0.05	*0.858*	0.40	*0.121*
PCR (occipital)						
All	0.46	***0.001***	0.37	***0.006***	0.41	***0.002***
TDC	0.00	*1.000*	0.06	*0.772*	−0.05	*0.836*
ASD	0.63	***0.015***	0.44	*0.112*	0.63	***0.017***
SPD	0.41	*0.117*	0.19	*0.488*	0.54	***0.029***

The bilateral tracts demonstrating significant combined-group associations with the inattention measure of the Sensory Profile are displayed. Correlation coefficients and p values are displayed for these tracts, along with combined group unilateral associations, individual group bilateral associations, and individual group unilateral associations.

**Figure 8:**
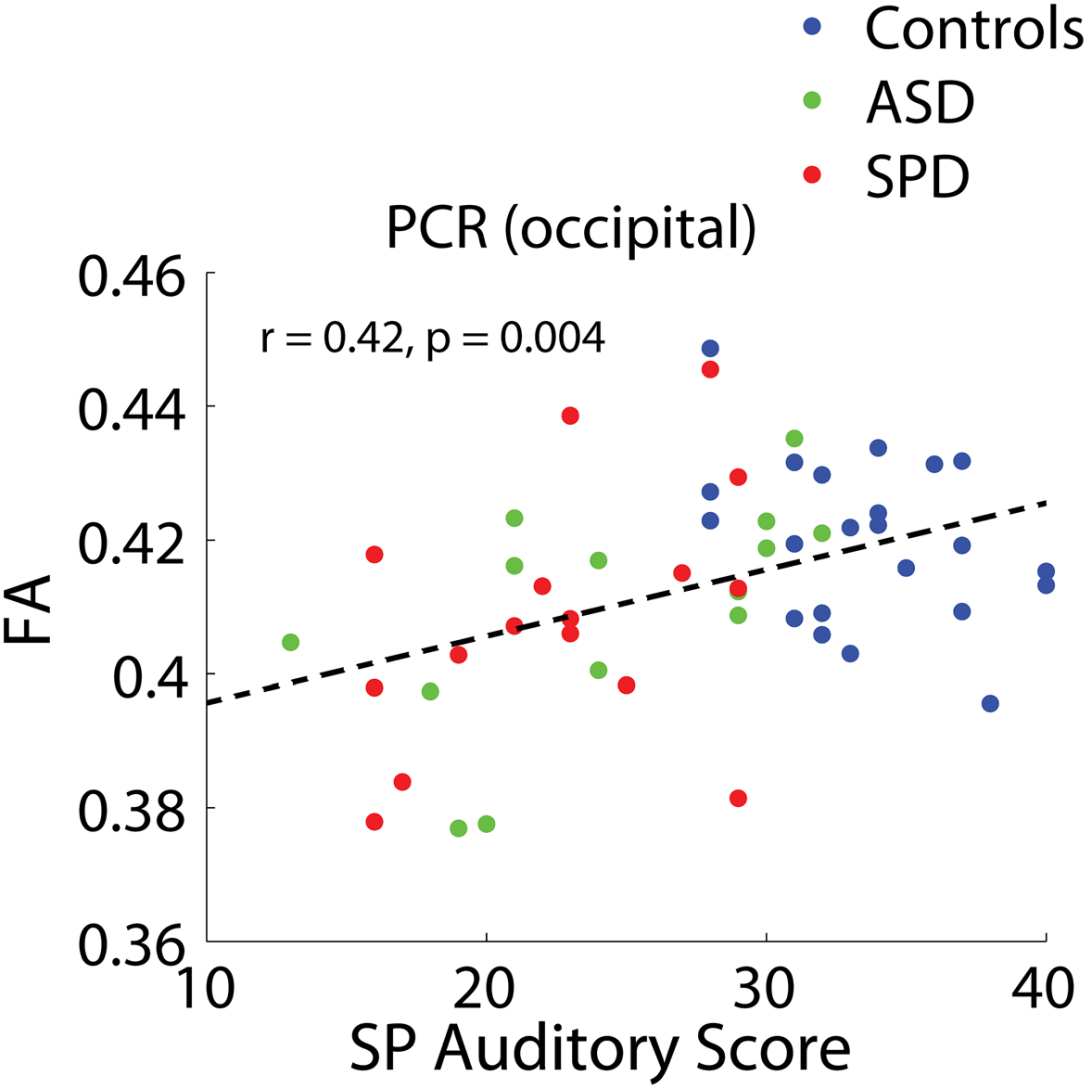
Combined-group associations between tract connectivity and the auditory measure of the Sensory Profile. The bilateral tract demonstrating significant associations between FA and the auditory measure of the Sensory Profile after FDR correction are displayed. PCR (occipital): r = 0.42, p = 0.002. Results of unilateral and individual group correlations are displayed in Table 10.

Significant combined-group correlations were found between the auditory factor of the Sensory Profile and the bilateral PCR (occipital) (r = 0.42, p = 0.004) (Figure 8). There is no strong evidence of lateralization for the combined groups (Table 10). The association between this auditory measure and FA in the PCR is consistent with our previously published findings in the combined SPD and TDC cohorts [16].

**Table 10:** Associations between tract connectivity and the auditory factor of the Sensory Profile, for significantly associated tracts.

	r - bilateral	*p - bilateral*	r - left	*p - left*	r - right	*p - right*
PCR (occipital)						
All	0.42	***0.004***	0.33	***0.017***	0.37	***0.007***
TDC	−0.33	*0.129*	−0.20	*0.353*	−0.27	*0.216*
ASD	0.60	***0.023***	0.38	*0.185*	0.62	***0.021***
SPD	0.41	*0.114*	0.20	*0.465*	0.49	*0.056*

The bilateral tracts demonstrating significant combined-group associations with the inattention measure of the Sensory Profile are displayed. Correlation coefficients and p values are displayed for these tracts, along with combined group unilateral associations, individual group bilateral associations, and individual group unilateral associations.

The combined groups did not demonstrate significant correlations between FA in the 15 investigated tracts and PRI, VCI, or the other subscores of the Sensory Profile after correction for multiple comparisons.

### 3.5. Tract overlap

**Figure 9:**
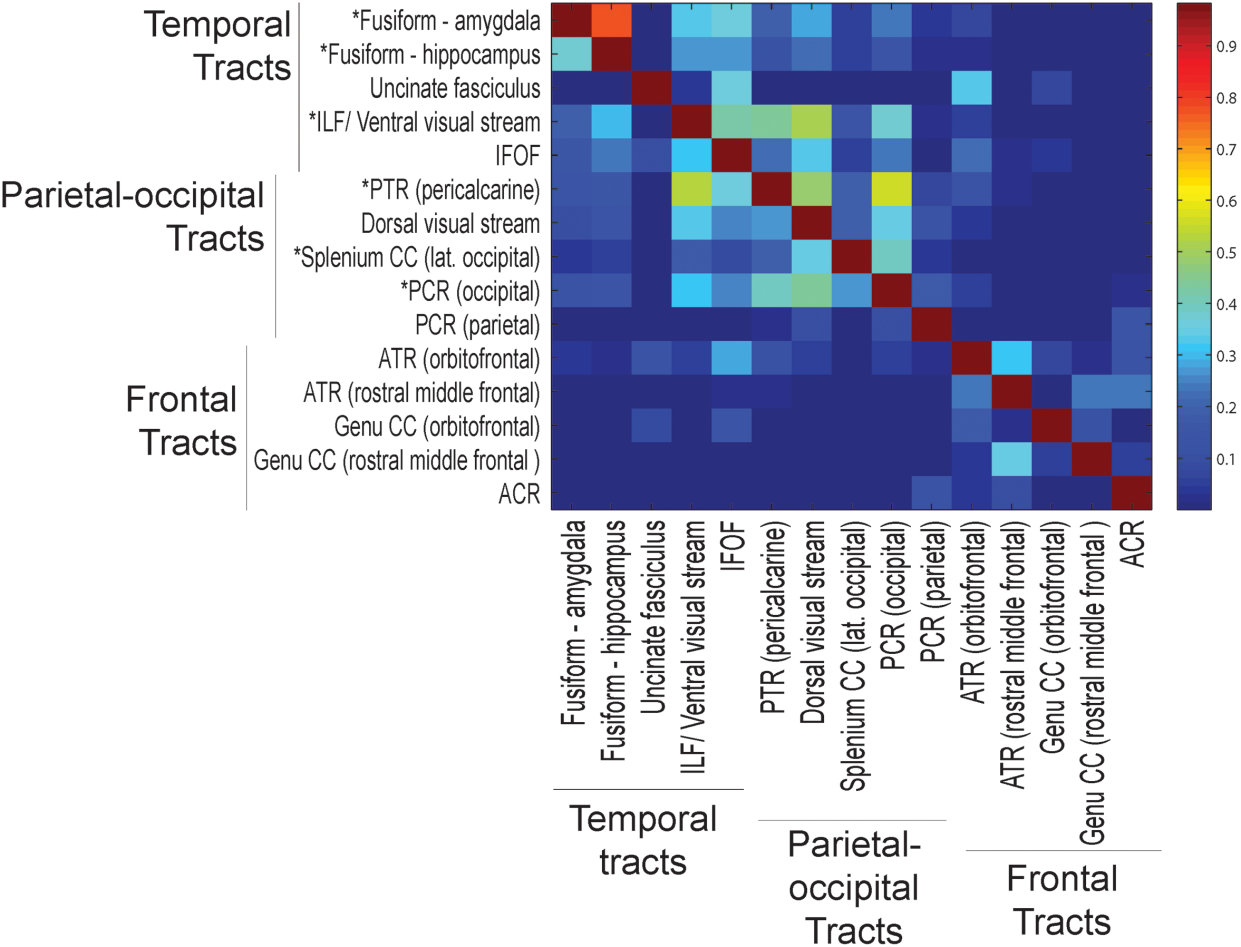
Average fraction of tract overlap. Color intensity corresponds to the subject average of the fraction of the voxels of the tracts on the vertical axis that are contained within the tracts on the horizontal axis. Tracts that are more than one-third contained in any other tract are indicated by an asterisk on the vertical axis.

Some tracts demonstrated a significant fraction of shared voxels. Figure 9 depicts the average fraction of each tract’s voxels that are shared with every other investigated white matter tract. In the most extreme case, a subject average of 77% of the fusiform - amygdala tract voxels were contained within the fusiform - hippocampus tract. There were also significant spatial overlaps within the delineated parietal-occipital tracts, and between some parietal-occipital and temporal tracts. These results demonstrate that some tracts were not completely independent in the group difference results above. Sections of the fusiform-amygdala tract that were independent of the fusiform-hippocampus, and sections of the fusiform-hippocampus that were independent of the fusiform-amygdala tract, were separately assessed for group differences. Neither of the independent sections of these tracts demonstrated statistically significant group differences, suggesting that shared voxels drive the observed differences between the ASD cohort and controls.

## Discussion

This study is the first to investigate white matter connectivity of both children with SPD and children with ASD relative to typically developing children. Diffusion MR fiber tracking was employed for the hypothesis-driven identification of specific white matter tracts. The results suggest both overlapping and divergent white matter microstructural pathology affecting the two clinical cohorts, with tracts traditionally associated with social emotional processing being significantly affected for the ASD cohort relative to TDC, but relatively unaffected in SPD. While both the ASD and the SPD participants demonstrate white matter pathology in the sensory processing regions of the dorsal visual stream and the posterior corona radiata, only the SPD cohort demonstrates statistically significant differences in the splenium of the corpus callosum relative to the TDC cohort. These findings extend previous research using DTI in autism cohorts to include concurrent analysis of children that exhibit sensory processing differences, but not the language and social deficits that characterize a full ASD diagnosis.

While the most extensive white matter alterations in the SPD subjects are observed in the parieto-occipital tracts, which subserve auditory, tactile, and visual perception and integration, this cohort demonstrates trends towards decreased connectivity compared to TDC in most measured tracts. It is also worth specific comment that, while both the SPD and ASD cohorts were affected in these fundamental sensory processing tracts, the FA in all but one of these tracts trended lower for the SPD subjects relative to the ASD subjects. This difference may reflect the prominence of abnormal sensory related behaviors, which is an inclusion criterion for SPD group membership, whereas, in general, children with ASD are primarily characterized by profound social communication deficits. In this sample, 65% of children with ASD scored in the definite difference (DD) range (>2 standard deviations from the mean) on the Sensory Profile Total Score and 57% were in the DD range for the Auditory Processing Score. While many children with ASD have auditory, tactile and visuomotor processing challenges, these deficits are not as ubiquitous as in our SPD cohort. Our findings further suggest that sensory-based behavioral deficits in both groups may be predicated on atypical conduction of information from unimodal to multimodal integration regions as well as inefficient transfer of information between hemispheres via the corpus callosum for the SPD group.

Perhaps the most striking finding is that, relative to the control group, the ASD cohort shows reduced structural connectivity in the fusiform gyrus connections to the amygdala and hippocampus, whereas children with SPD do not. These white matter pathways are thought to facilitate facial emotional processing, a core feature of autism and the domain of clinical divergence for ASD versus SPD [29]. In fact, a recent study reports that infants later diagnosed with autism show reduced attention to essential facial information with declining direct eye gaze as early as 2–6 month of age [30]. The neuroanatomy of facial emotion processing has been intensively studied with repeated implication of the amygdala-fusiform system. Individuals with ASD have been reported to have less activation of subcortical regions including the amygdala and fusiform gyrus during subliminal emotional face processing, a lack of expected volumetric correlation between the amygdala and fusiform gyrus, as well as behavioral deficits in face recognition that negatively correlate with left fusiform cortical thickness [31], [32]. In a DTI based analysis of adolescents and adults with autism, the right hippocampal fusiform tract was suggested to have smaller diameter axons corresponding with slower neural transmission, which was thought to lead to secondary changes in the left amygdala-fusiform and hippocampal-fusiform pathways [16]. By contrast, a diffusion fiber tractography study of individuals with Williams syndrome (7q11.23 deletion) are reported to show elevations of FA in fusiform tracts [33]. Individuals with Williams Syndrome show a social phenotype that is in some ways opposite to the autism phenotype with increased attention to faces and abundant social interest and drive. It is thus worthwhile to consider social cognition, or facial emotion recognition specifically, as a continuous trait that might map directly to connectivity of the fusiform tract to limbic structures.

There are however additional farther reaching implications for fusiform connectivity disruptions with regard to language development. A theoretical model of audiovisual affective speech perception begins with input to primary auditory and visual cortex [34]. The *input module* feeds information to the fusiform gyrus as well as the *integration module* of the superior and middle temporal cortex. The primary sensory cortices as well as the fusiform gyrus are reciprocally connected to the amygdala and insula, which comprise the *emotion module* that guides emotional relevance and may facilitate the rapid recruitment of limbic brain regions by visual inputs. Additional contextual information is brought in through connections with the *memory module*, including the hippocampus and parahippocampal gyrus. This framework is useful in considering how autism social communication deficits may map to neuroanatomic networks.

In addition to the fusiform connections, our ASD group was found to have reduced FA in the ILF and the IFOF. This is in line with previous reports, although there is considerable variability in the literature, likely resulting from group heterogeneity in terms of symptom variability, severity, and age of cohort [8], [35]–[38]. The ILF, or inferior longitudinal fasciculus, has been shown to directly connect the occipital cortex to the anterior temporal lobe and the amygdala. The IFOF originates in the visual cortex runs medially to the ILF and directly connects to the inferior frontal and dorsolateral frontal cortex. In a study involving children with visual perceptional impairment, decreased FA of the ILF correlated with impaired object recognition [39]. The IFOF likely overlaps spatially and functionally with the ILF, and is thought to be a tract that is relatively new evolutionarily due to its absence in animal brains [40]. In a large lesion-based study population, the right IFOF in particular is implicated in rapid recognition of emotional facial expressions [41]. The finding of significantly reduced connectivity in ASD of the ILF and IFOF is in concordance with the reduced connectivity seen specifically in the fusiform connections. What is most revealing is the relative preservation of these connections in our SPD cohort. Clearly additional investigation to understand the relationship between the speed of information transmission in these tracts as well as the behavioral correlates of altered connectivity is warranted. However, these findings suggest a role for neuroimaging in understanding the neural mechanisms that differentiate children with a variety of domain specific deficits, including basic sensory processing and social emotional processing. The role of development and therapeutic interventions on these systems remains an open and important question to explore in these clinical cohorts as it is unclear whether these findings are primary or represent a consequence of aberrant tract remodeling predicated on less practice of these skills from early infancy.

As can be seen from the group comparison figures, while there are clear and statistically significant group differences, there is also considerable overlap in the measurements from tracts across all three groups: ASD, SPD, and TDC. This highlights the importance of a new direction for cognitive and behavioral research based on the investigation of abilities as a continuous measure across children rather than split by exceedingly broad and overlapping clinical labels, a concept which has been formalized in the Research Domain Criteria (RDoC) Project [42]. It is in this context that we frame our investigation of associations between cognitive measures and tract connectivity across all three study groups. The fusiform-amygdala and fusiform-hippocampus tracts are the only two (out of 15) tracts to demonstrate significant associations with the social component of the SCQ across groups with correction for multiple comparisons (Figure 6, Table 8). Further investigation of laterality in these two tracts revealed that these associations are significantly right-lateralized (Table 8), an observation which is consistent with the Conturo et al. (2008) [16] finding of primary right-lateralized effects in these two tracts for ASD subjects. It is important to note that the associations between connectivity in these two tracts and the SCQ-social measure were not significant on an individual group basis. However, the individual group correlations all trended in the same (negative) direction as the combined-group correlations (Table 8). Connectivities (FA) in the optic radiation and PCR (occipital) were found to be significantly correlated with WMI after FDR correction in the combined groups. Investigation of these associations in the individual cohorts revealed strong associations with WMI for the SPD cohort bilaterally in both of these tracts. The ASD cohort alone also demonstrates significant or trend-level associations with WMI and connectivity in these tracts. Though the optic radiation and PCR (occipital) were the only two tracts that demonstrated significant associations with WMI after FDR correction, many of the other investigated tracts (including frontal, temporal, and parietal-occipital tracts) demonstrated trend-level associations. This is consistent with reports from prior DTI studies of WMI that have found widespread associations of WMI with white matter connectivity [43], [44]. While our findings of significant associations between connectivity in the PCR (occipital) and the Sensory Profile auditory and inattention measures are consistent with our prior findings [16], we did not find significant associations with the other Sensory Profile measures after correction for multiple comparisons.

There are important limitations to note for this study, which should motivate further investigation. First, we have not determined an optimal method for characterizing the sensory subtypes and distinguishing between hypo- or hyper-sensory sensitivity, nor do we have sufficient power in this study for sensory subtype group analysis. We and many sensorimotor based researchers are working to develop a phenotyping tool that maps to specific white matter tracts, and we hope to identify and characterize separate constructs of sensory deficits in larger cohorts going forward. Second, tract overlap exists in our results. A significant portion of the amygdala-fusiform tract is contained within the hippocampal-fusiform tract. In addition, the ILF is partially contained within the dorsal visual stream and the PTR is partially contained within the PCR. Despite these spatial overlaps, the group difference results are not identical between overlapping tracts and provide separately valuable information about structural connectivity in these subjects. Additional connectivity analysis, both structural and functional, will shed additional light on specific regional contributions to the neural underpinnings of sensory and emotional processing differences. Our investigation is also limited in generalizability, as all of the subjects were boys between the ages of 8 and 12 years in an effort to limit developmental confounds in this small sample. The PRI scores of the ASD cohort were significantly lower than that of the SPD and TDC cohorts; however, the most important group differences in structural connectivity between ASD and controls remained statistically significant after regressing out the effect of PRI. Further research is therefore needed to determine whether these findings generalize to other ages, genders, and intellectual abilities.

Future research will include investigation of functional connectivity using resting state fMRI and magnetoencephalography (MEG). The ROIs used to determine structural connectivity in this study can be used to assess differences in functional connectivity between these same regions, with functional coupling hypothesized to be reduced where decreased structural connectivity was found in this work. While prior studies have found relationships between functional connectivity and white matter volume in ASD [45]–[47], there have been no reported associations between functional connectivity and DTI measures in SPD.

We hope that by utilizing larger sample sizes and direct assessment of auditory, tactile, visuomotor processing, we will be able to gain a deeper understanding of how neural circuitry differences map to clinically relevant challenges for individual children. The ultimate goal of this and future work is to guide personalized treatments ranging from behavioral interventions and targeted psychopharmacology to cognitive training using child-friendly video game platforms.

## Supporting Information

Figure S1 **Example ROIs for fiber tracking of the homotopic visual tract through the splenium of the corpus callosum.** Displayed is an axial slice from the FA image of a representative subject with overlaid ROIs for probabilistic fiber tractography. The seed mask is the grey-white matter boundary of the left lateral occipital cortex, and contains voxels from which 2000 streamlines each are initiated. The termination mask is the grey-white matter boundary of the right lateral occipital cortex, and causes streamlines to terminate upon encountering the mask. The termination mask and the corpus callosum are both used as waypoint masks, indicating that streamlines need to pass through the corpus callosum and reach the termination mask in order to be retained. The exclusion mask is the union of the grey-white matter boundaries of all other cortical regions, and causes streamlines that encounter these voxels to be excluded. The displayed resultant tract is the result of probabilistic tractography under the previously described constraints and a subsequent streamline and FA threshold.
(TIFF)

Figure S2 **Group differences of MD in all tracts. Units of diffusivity are in mm^2^/sec.** Asterisks indicate significant differences based on two-tailed permutation tests with FDR correction for 15 comparisons.
(TIFF)

Figure S3 **Group differences of RD in all tracts. Units of diffusivity are in mm^2^/sec.** Asterisks indicate significant differences based on two-tailed permutation tests with FDR correction for 15 comparisons.
(TIFF)

Figure S4 **Group differences of AD in all tracts. Units of diffusivity are in mm^2^/sec.** Asterisks indicate significant differences based on two-tailed permutation tests with FDR correction for 15 comparisons.
(TIFF)

Table S1 **Group differences of FA between the ASD and SPD cohorts in ASD-affected temporal tracts.** P values are derived from one-tailed permutation tests for SPD FA > ASD FA. Bolded p values indicate significant differences after FDR correction.
(XLSX)

Table S2 **Group differences of FA in all tracts, excluding SPD subjects with SCQ>15.** P values are derived from one-tailed permutation tests for TDC FA > SPD FA. Bolded p values indicate significant group differences after FDR correction.
(XLSX)
