# Supplementary figures and images for: Autism and Sensory Processing Disorders: Shared White Matter Disruption in Sensory Pathways but Divergent Connectivity in Social-Emotional Pathways
Source: PLoS One. 2014 Jul 30;9(7):e103038. doi: 10.1371/journal.pone.0103038 (PMC4116166; doi:10.1371/journal.pone.0103038)

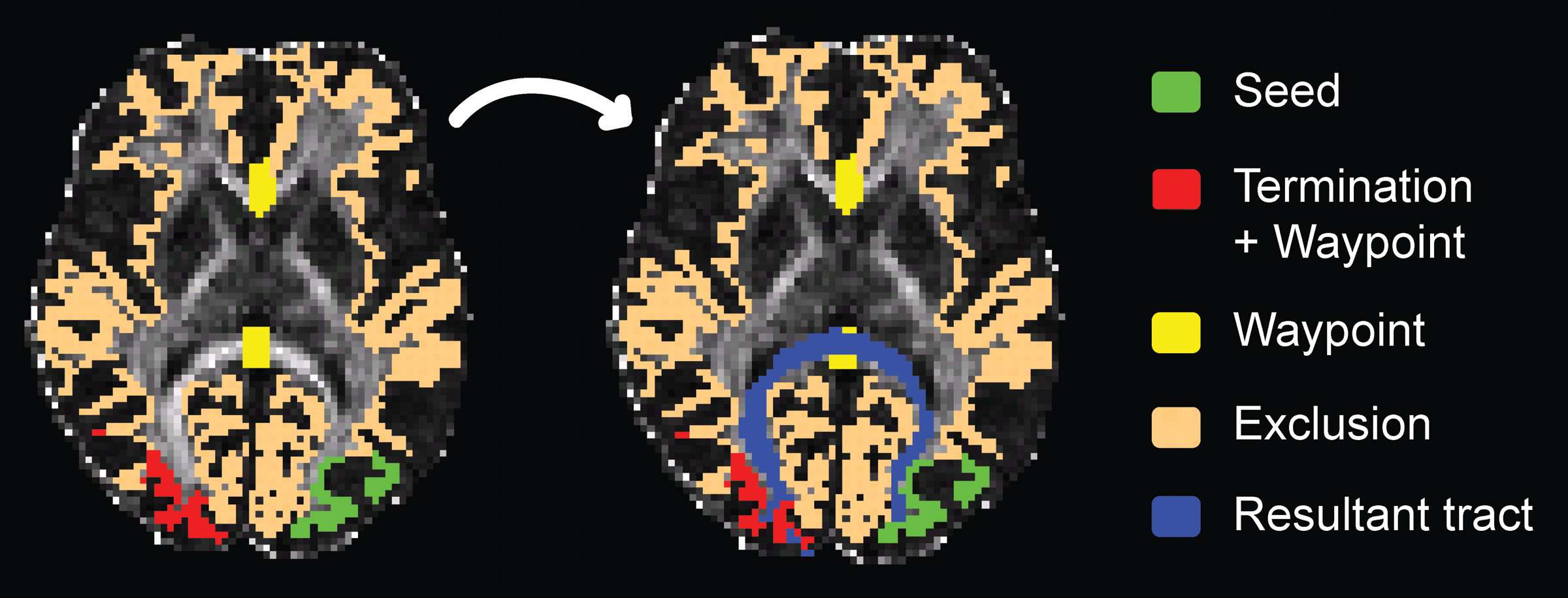

Supplement: Figure S1 — Example ROIs for fiber tracking of the homotopic visual tract through the splenium of the corpus callosum. Displayed is an axial slice from the FA image of a representative subject with overlaid ROIs for probabilistic fiber tractography. The seed mask is the grey-white matter boundary of the left lateral occipital cortex, and contains voxels from which 2000 streamlines each are initiated. The termination mask is the grey-white matter boundary of the right lateral occipital cortex, and causes streamlines to terminate upon encountering the mask. The termination mask and the corpus callosum are both used as waypoint masks, indicating that streamlines need to pass through the corpus callosum and reach the termination mask in order to be retained. The exclusion mask is the union of the grey-white matter boundaries of all other cortical regions, and causes streamlines that encounter these voxels to be excluded. The displayed resultant tract is the result of probabilistic tractography under the previously described constraints and a subsequent streamline and FA threshold. (TIFF) [file pone.0103038.s001.tiff]

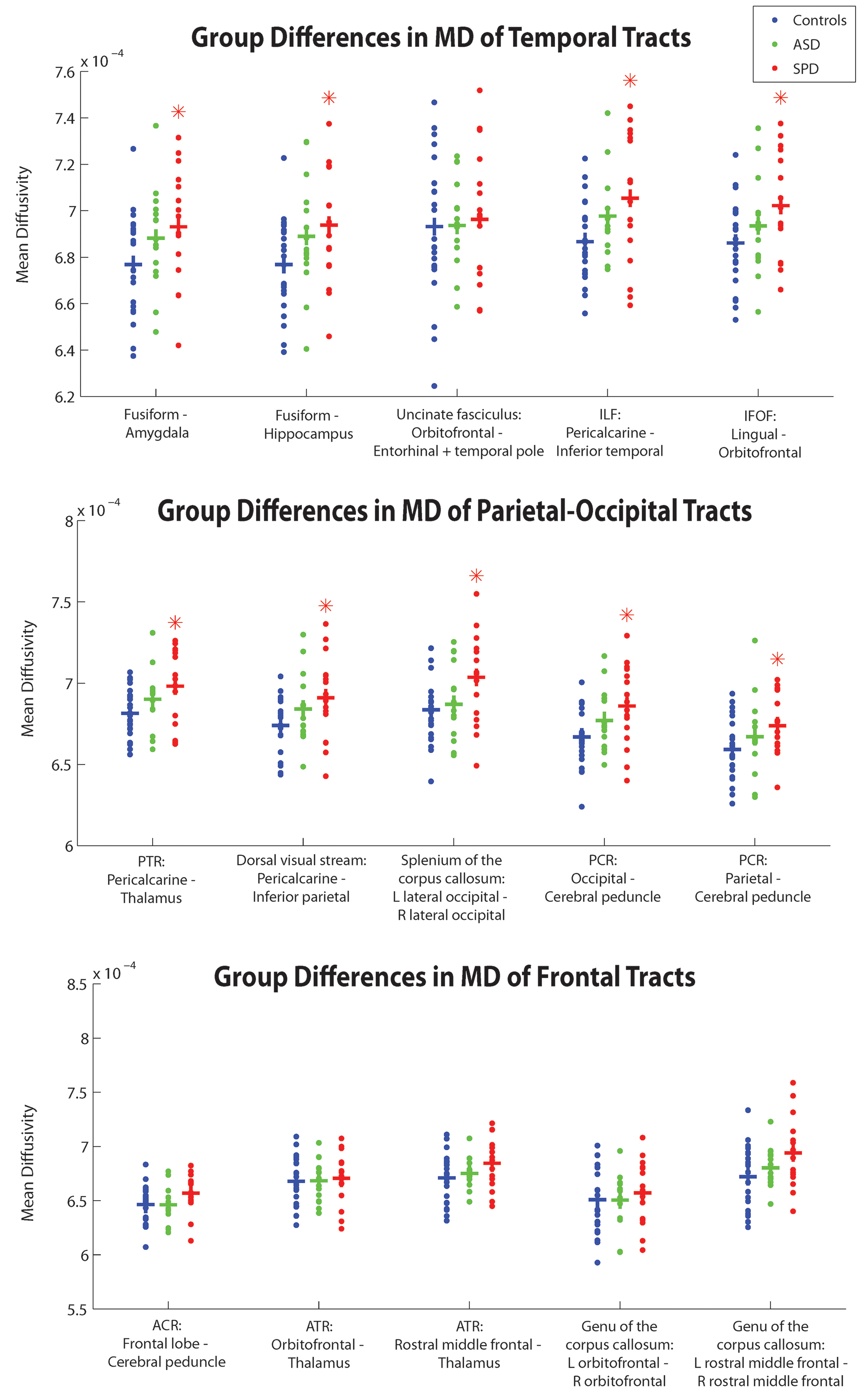

Supplement: Figure S2 — Group differences of MD in all tracts. Units of diffusivity are in mm2/sec. Asterisks indicate significant differences based on two-tailed permutation tests with FDR correction for 15 comparisons. (TIFF) [file pone.0103038.s002.tiff]

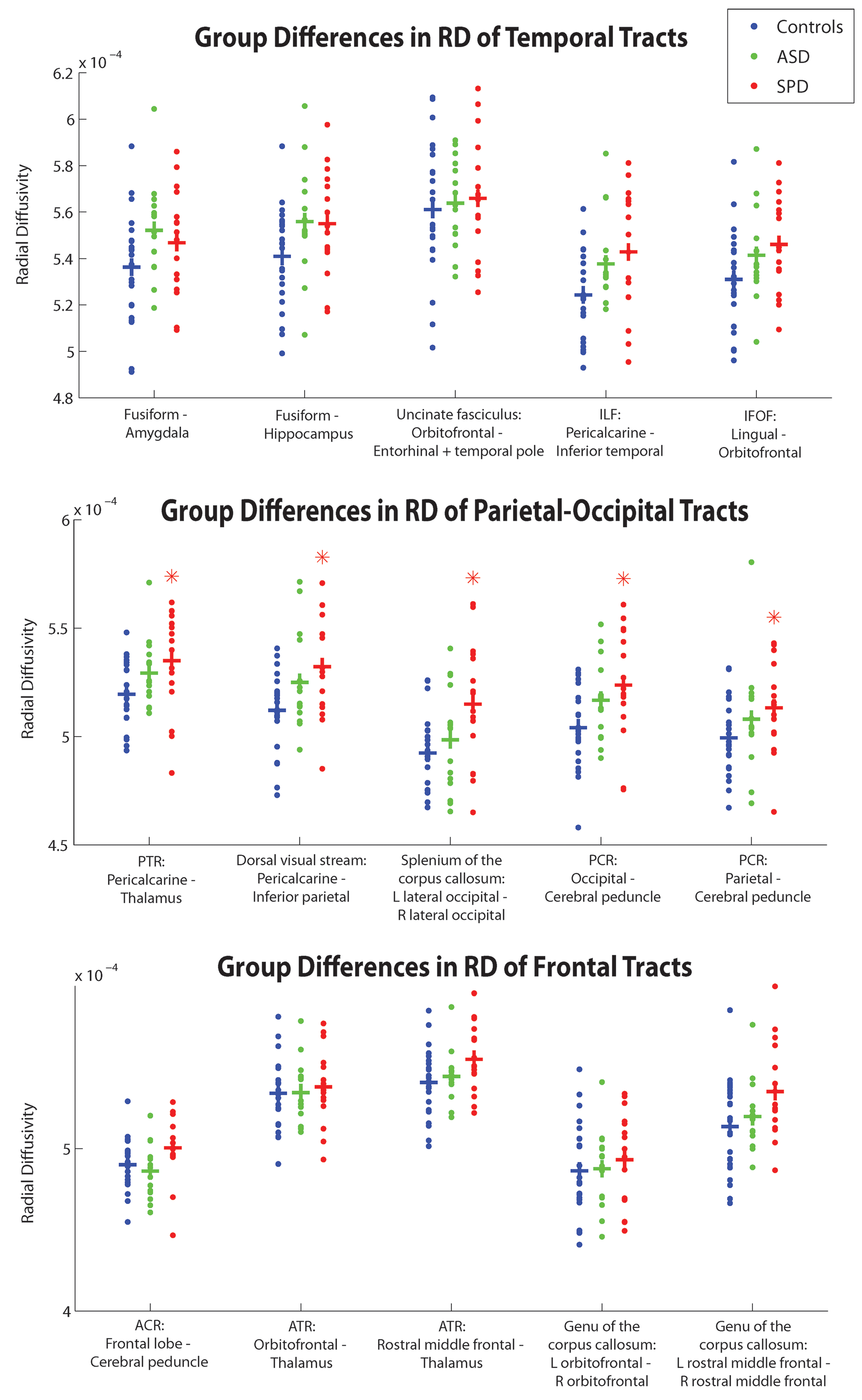

Supplement: Figure S3 — Group differences of RD in all tracts. Units of diffusivity are in mm2/sec. Asterisks indicate significant differences based on two-tailed permutation tests with FDR correction for 15 comparisons. (TIFF) [file pone.0103038.s003.tiff]

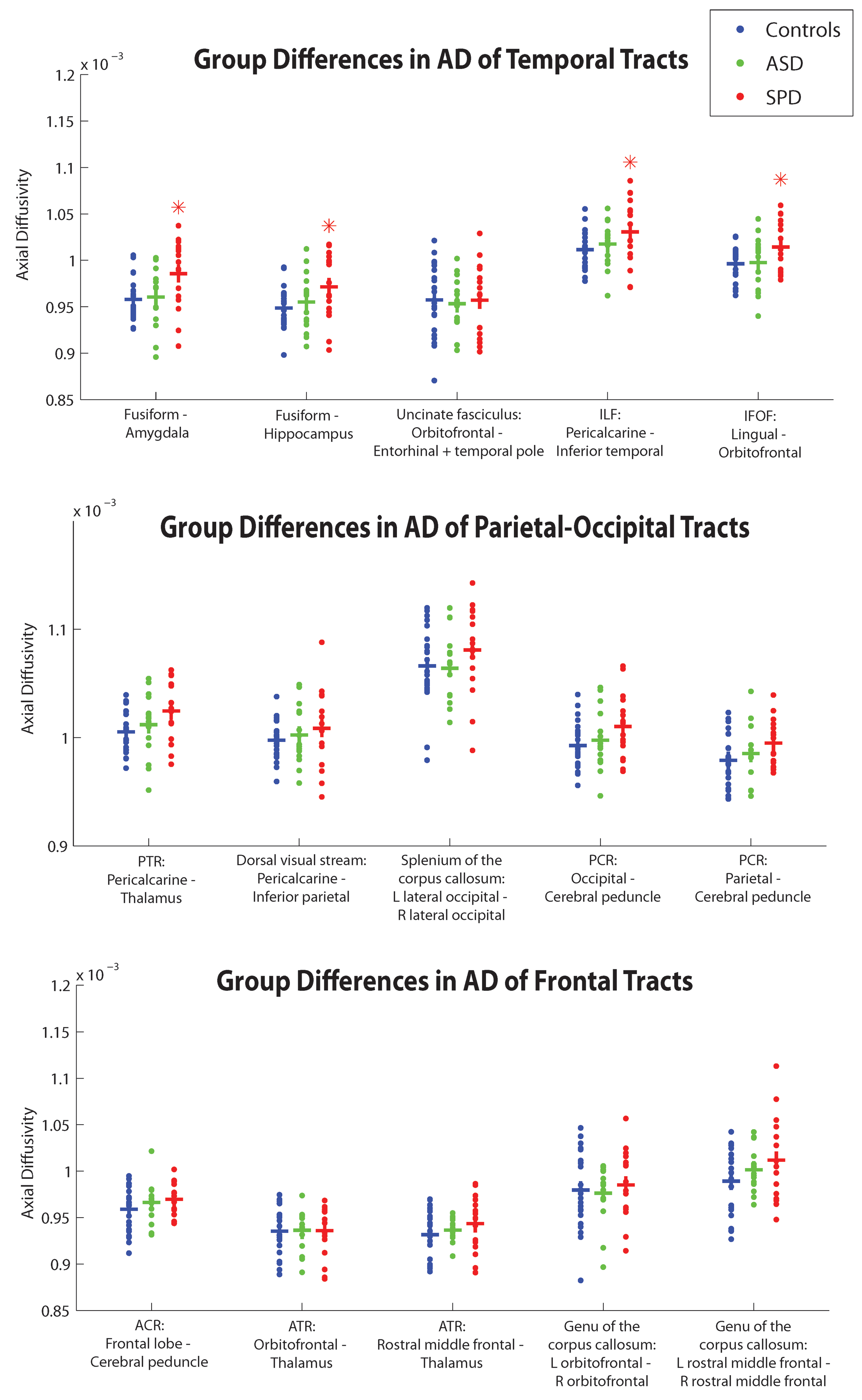

Supplement: Figure S4 — Group differences of AD in all tracts. Units of diffusivity are in mm2/sec. Asterisks indicate significant differences based on two-tailed permutation tests with FDR correction for 15 comparisons. (TIFF) [file pone.0103038.s004.tiff]
